# Increased Transmission of Mutations by Low-Condition Females: Evidence for Condition-Dependent DNA Repair 

**DOI:** 10.1371/journal.pbio.0060030

**Published:** 2008-02-12

**Authors:** Aneil F Agrawal, Alethea D Wang

**Affiliations:** Department of Ecology and Evolutionary Biology, University of Toronto, Toronto, Ontario, Canada; Duke University, United States of America

## Abstract

Evidence is mounting that mutation rates are sufficiently high for deleterious alleles to be a major evolutionary force affecting the evolution of sex, the maintenance of genetic variation, and many other evolutionary phenomena. Though point estimates of mutation rates are improving, we remain largely ignorant of the biological factors affecting these rates at the individual level. Of special importance is the possibility that mutation rates are condition-dependent with low-condition individuals experiencing more mutation. Theory predicts that such condition dependence would dramatically increase the rate at which populations adapt to new environments and the extent to which populations suffer from mutation load. Despite its importance, there has been little study of this phenomenon in multicellular organisms. Here, we examine whether DNA repair processes are condition-dependent in Drosophila melanogaster. In this species, damaged DNA in sperm can be repaired by maternal repair processes after fertilization. We exposed high- and low-condition females to sperm containing damaged DNA and then assessed the frequency of lethal mutations on paternally derived X chromosomes transmitted by these females. The rate of lethal mutations transmitted by low-condition females was 30% greater than that of high-condition females, indicating reduced repair capacity of low-condition females. A separate experiment provided no support for an alternative hypothesis based on sperm selection.

## Author Summary

A variety of evolutionary phenomena are affected by the rate at which mutations enter a population and how those mutations are distributed amongst individuals. Although it is typically assumed that mutations occur randomly among individuals, this may not be the case. Individuals in poor condition may experience elevated mutation rates if they are more prone to experiencing DNA damage or are less able to repair such damage. Using the fruit fly Drosophila melanogaster, we tested whether individuals in poor condition had a reduced capacity to efficiently repair mutagen-induced DNA damage. Consistent with the prediction, we recovered approximately 30% more mutations from low-condition individuals than from high-condition individuals in two separate experiments. Such condition dependence in mutation rate may cause populations to carry considerably heavier loads of deleterious mutations than otherwise expected.

## Abstract 3

When female flies were inseminated with mutagenized sperm, the resulting offspring were 30% more likely to carry a lethal mutation if the female was in low rather than high condition.

## Introduction

Germ-line mutation is the ultimate source of heritable variation, but the vast majority of new mutations affecting fitness are deleterious. The unremitting presence of deleterious mutations causes a reduction in mean fitness, a phenomenon known as mutation load. Mutation load can be substantial even if individual mutations are of small effect and are held at low frequencies by natural selection. For example, classic theory [1] predicts that mutation load will reduce mean fitness by more than 60% if there is just one deleterious germ-line mutation per genome per generation.

The constant influx of deleterious mutations may pose a serious challenge to natural populations [2]. Mutation load can accelerate the extinction of endangered species [3,4] and may be an important public health concern in humans [5]. Large mutation loads have been invoked as a possible explanation for a wide variety of other phenomena, such as the maintenance of genetic variation [6], the evolution of specialization [7,8], the evolution of outcrossing [9–11], and the evolution of sexual reproduction [12–15].

Mutation rate (*U*) is the most important factor determining the magnitude of mutation load. However, estimates of the mutation rate vary over two to three orders of magnitude [16–20]. While much of this variance may be due to measurement error (especially in earlier studies), some of this variance likely has real biological causes [17,21–23]. Of special interest is the possibility that variance in mutation rate arises from individual variation in condition, because individuals of low condition may have elevated rates of mutation. In a stable environment, condition dependence of the mutation rate is expected to alter the mutation load [24] because of the positive feedback loop it creates: individuals with an excess of deleterious alleles tend to be in low condition and so experience a high mutation rate. (Interestingly, the mutation loads of sexual and asexual populations are affected very differently by condition dependence [24].) Condition dependence is also expected to accelerate adaptation to new stressful environments if the mutational input is elevated under poor condition [25,26].

Although mutation rate is often treated as though it is constant and nonplastic, there is no compelling reason to believe this should be true. Condition dependence is common among other traits, including recombination, another “genomic” trait [27–29]. Moreover, there is evidence that mutation rate varies across environments in some unicellular organisms [30–32]. However, there are reasons to question whether patterns observed in unicellular organisms apply to multicellular organisms. First, the unicellular organisms cited above are predominantly asexual, and theory [33,34] predicts that facultative elevation of mutation rate in response to stress is more likely to evolve for adaptive reasons in asexual species than in sexual species. Second, unicellular organisms may be particularly sensitive to environmental effects on DNA processes simply because they are unicellular. Nonetheless, it is reasonable to predict that mutation rates are also condition-dependent in multicellular organisms, though the reasons may be different from those in unicellular organisms. For instance, condition dependence may occur because maintaining DNA with perfect fidelity is a costly enterprise and low-condition individuals are less able to pay this cost. Despite the potentially important consequences of this phenomenon, there has been little effort to look for evidence of condition dependence in multicellular organisms.

Mutation rate is a function of two factors: (1) the rate at which DNA damage occurs and (2) an organism's ability to repair that damage. Condition dependence in mutation rate is expected if either of these factors is condition-dependent. Using Drosophila melanogaster, we tested whether individuals in low condition are less able to repair DNA damage without inducing a mutation. When lesions in the DNA occur, these must be repaired for the cell cycle to continue properly. Some repair pathways are conservative and do not result in mutation; other repair pathways are error-prone so that mutations are generated in the process of removing DNA lesions. Conservative pathways are thought to be more costly than error-prone pathways [35]. The premise of our experiment was simple: expose high- and low-condition individuals to damaged DNA and assess their ability to repair the DNA without introducing error.

**Figure 1:**
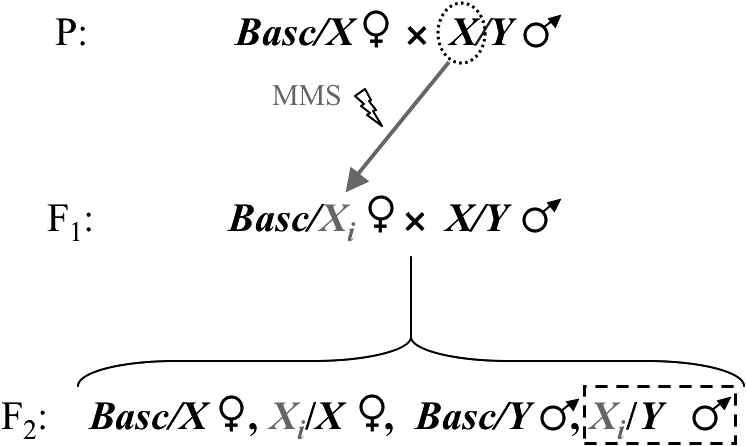
Crossing Design for Detection of Sex-Linked Recessive Lethal Mutations In the parental cross, high- or low-condition females of genotype *Basc/*X were mated to wild-type males who had been mutagenized with MMS. From each mother, four to ten F_1_ daughters were placed in individual vials to lay eggs. Each of these daughters carried a paternally inherited (mutagenized) X chromosome, denoted as X*_i_* in grey. These females had been allowed to mate with their brothers, which means they could have mated to either *X/Y* or *Basc*/Y males though offspring arrays indicate that it was usually the former as depicted here. Regardless of the F_1_ male, 25% of the F_2_ offspring are expected to be X*_i_* / Y (wild-type) males (shown in dashed box). However, if there is a recessive lethal (or near lethal) on X*_i_*, then X*_i_* / Y males will be absent or very rare. We attempted to classify each X*_i_* as carrying a (near) lethal or as not doing so, depending on the likelihood of obtaining the observed frequency of X*_i_ / *Y males when the expected frequency was 25% (no lethal) relative to the likelihood when the expected frequency was 2.5% (near lethal). If there was not sufficient evidence from the distribution of F_2_ progeny to classify an X*_i_* in either of these two categories, then that X*_i_* was excluded from further analysis. See Materials and Methods for details.

One cannot simply expose flies to the same mutagen treatment because high- and low-condition individuals might respond differently (e.g., by eating or absorbing different amounts of the mutagen) such that the level of damage differs between the treatments. To circumvent this difficulty, we took advantage of the maternal repair system in D. melanogaster. When males are mutagenized, DNA damage in sperm persists because there is little, if any, postmeiotic repair in males [36,37]. However, premutational DNA lesions can be repaired after fertilization by maternal repair proteins. For example, Vogel et al [37] mated standard males that had been mutagenized with methyl methanesulfonate (MMS) to either wild-type or repair-deficient (*Mei-9* mutant) females. Repair-deficient females produced daughters carrying recessive lethal mutations on their *paternally* derived (i.e., mutagen-exposed) X chromosomes almost eight times more frequently than did wild-type females. This result indicates repair-deficient females were less able to repair DNA damage on chromosomes coming from mutagenized sperm without producing a mutation. We used a similar design comparing high- and low-condition females rather than wild-type and repair-deficient females. Specifically, we used a larval diet manipulation to create high- and low-condition females that were genetically wild type with respect to DNA repair genes. These females were mated to standard mutagenized males; daughters were then screened for recessive lethals on the paternally inherited X chromosome (Figure 1).

As reported below, low-condition females transmitted more of these sex-linked recessive lethals (SLRLs) than did high-condition females. An alternative interpretation to condition-dependent repair is condition-dependent sperm selection. In this scenario, heavily damaged sperm would be less likely to fertilize eggs in high-condition females than in low-condition females. We examined this possibility by doing a separate sperm competition experiment in which we measured selection against mutagenized sperm in both high- and low-condition females. There is no evidence that selection against mutagenized sperm is stronger in high-condition females.

## Results

### Transmission Rate of Lethal-Bearing Paternal X Chromosomes

A diet manipulation was used to produce flies of high and low condition. Females emerging from the low-condition treatment tended to be visibly smaller than females from the high-condition treatment, but all flies were well within the normal range of body sizes observed in typical fly cultures. Both high- and low-condition females were mated to males that had been reared under standard conditions and then mutagenized with alkylating agent MMS. As expected, the diet manipulation affected condition: females from the low-condition treatment produced approximately 32% fewer offspring than females from the high-condition treatment (*F*_1,646_ = 82.0, *p* < 0.0001).

Averaging across all of Experiment 1, we found that approximately 15% of paternally derived (i.e., mutagenized) X chromosomes carried lethal (or near-lethal) mutations. This frequency is consistent with other studies using a similar dose of this mutagen and is about two orders of magnitude greater than the spontaneous rate [38]. Our primary interest is whether a mutagenized X chromosome was more likely to eventually harbor a lethal mutation if it passed from a sperm into an egg in a low-condition female rather than a high-condition female. Such a pattern would be expected if low-condition females were more likely than high-condition females to employ error-prone pathways to repair damaged DNA from sperm. The frequency at which high- and low-condition females transmitted SLRL mutations from their mutagenized mates to their offspring is given in Table 1. We observed a higher frequency of lethal-bearing X chromosomes being transmitted by low-condition females than by high-condition females. A randomization test revealed that we were unlikely to observe this large a difference by chance (*n* = 552, *p* = 0.04). Averaging over both blocks of Experiment 1, the rate at which low-condition females transmitted lethal-bearing X chromosomes (0.159) was approximately 28% higher than the rate at which high-condition females transmitted lethal-bearing X chromosomes (0.124), i.e., 0.159/0.124 = 1.28.

**Table 1:**
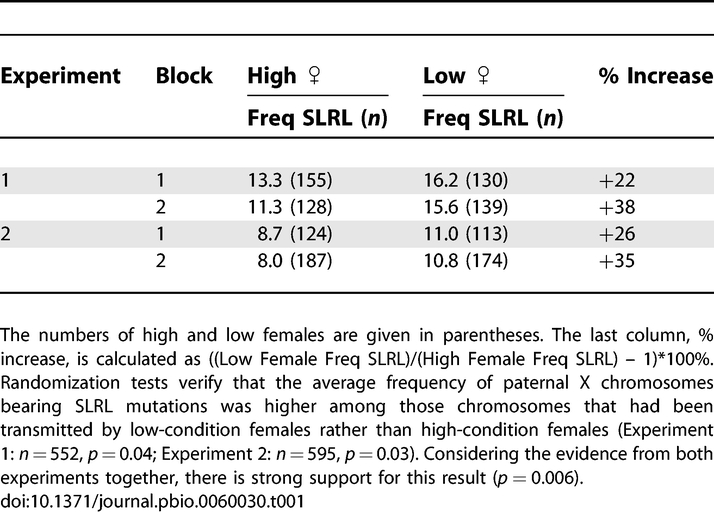
Average Frequency of X Chromosomes Bearing Sex-Linked Recessive Lethal Mutations Recovered from High- and Low-Condition Females Mated to Mutagenized Males

We performed a second experiment similar to that described above except that females were mated individually to mutagenized males to prevent any effects of pre-copulatory sexual selection. As in Experiment 1, the diet manipulation in Experiment 2 affected condition: females from the low-quality diet treatment produced significantly fewer offspring than females from the high-quality diet treatment (*F*_1,851_ = 10.7, *p* < 0.001). In Experiment 2, the overall rate of SLRLs was approximately 0.10, a lower frequency than in Experiment 1 but within the normal range expected for this type of mutagenesis [38]. As in Experiment 1, there was a difference in the frequency of SLRLs transmitted by low- and high-condition females (low: 0.109; high: 0.083). Relative to the daughters of high-condition females, the daughters of low-condition females were approximately 31% more likely to harbor a lethal mutation on their paternally inherited X chromosome (*n* = 595, *p* = 0.03).

The data shown in Table 1 indicate that our results are consistent across both blocks of both experiments: low-condition females transmit lethal-bearing paternally derived X chromosomes at a higher rate than high-condition females. Considering the evidence from both experiments together by combining *p*-values [39] indicates this is a strongly significant effect (weighted Z = −2.53, *p* = 0.006).

### Selection against Mutagenized Sperm in Low- and High-Condition Females

It is possible that the results above could be due to sperm selection rather than DNA repair capacity. Sperm carrying more heavily damaged chromosomes might be less likely to successfully fertilize eggs in high-condition females than in low-condition females. In other words, it is possible that there is stronger selection against mutagenized sperm in high-condition females than in low-condition females. To test this possibility, we measured the siring success of mutagenized males and non-mutagenized males when mated to high- or low-condition females.

Females were first mated to standard males and then mated to either mutagenized or non-mutagenized males. We measured the proportion of offspring sired by the second male (*P_2_*), thus allowing for estimates of the *P_2_* abilities of mutagenized and non-mutagenized sperm against a standard competitor. Mean *P_2_* scores are shown in Table 2. Analysis of these data with a generalized linear mixed model revealed a significant negative effect of mutagenesis on *P_2_* score (*F*_1,18894_ = 9.92, *p* = 0.002), i.e., mutagenized sperm was less successful than non-mutagenized sperm. There was no significant effect of female condition (*F*_1,18894_ = 0.00, *p* = 0.96), and there was no significant interaction between female condition and whether sperm had been mutagenized (*F*_1,18894_ = 0.31, *p* = 0.58).

**Table 2:**
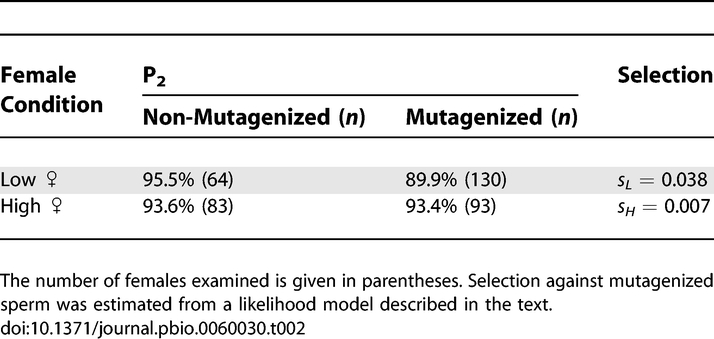
Competitive Ability of Non-Mutagenized and Mutagenized Sperm against a Standard Competitor, Measured as *P*_2_

In addition to the analysis described above, we performed a different likelihood analysis that allowed us to model the strength of selection against mutagenized sperm in low- and high-condition females as separate parameters that are more easily interpreted. Consistent with the previous analysis, this likelihood analysis indicated selection against mutagenized sperm in low-condition females (*s_L_* = 0.038) was considerably stronger than in high-condition females (*s_H_* = 0.007). Although *s_L_* was found to be significantly greater than *s_H_* in this analysis, these point estimates are primarily of heuristic value as this latter analysis ignores variation among individual females so that the model will tend to underestimate the uncertainty in the parameter estimates. Nonetheless, the results of both analyses show that whereas there is weak selection against mutagenized sperm, there is no indication that selection is stronger in high-condition females than in low-condition females—the evidence is in the opposite direction.

## Discussion

According to classic theory, deleterious alleles can have large effects on a population if the mutation rate is sufficiently large, i.e., on the order of *U* = 1. Though estimates of *U* have varied considerably, recent studies [16,17] employing modern techniques indicate that mutation rates are likely to be high enough to create large loads.

As estimates of the mutation rate continue to improve, we can begin to acknowledge and study the variation in this genomic property. It is well known that transposable element activity increases in response to extrinsic stress [40,41], but much less is known about variation in the rates of more traditional types of mutation. There is recent evidence that repair capacity and net mutation rate are temperature-dependent [23,42,43], though this may not be too surprising since both DNA and protein stability are sensitive to temperature. Some recent mutation-accumulation studies have found evidence that mutation rates vary between closely related species and even among lines of the same species [17,22]. One study reported that the mutation rate accelerated within a line over the duration of a long mutation-accumulation experiment [21]. The reasons for this variation are unclear. In some cases, the variation in mutation rate can be attributed to genetic differences among lines because all lines accumulated mutations in the same environment [22]. Even so, it is unknown whether the important genetic differences occur at loci that are directly involved in DNA replication and/or repair, or alternatively, whether genetic differences affecting condition indirectly lead to differences in mutation.

Despite the important consequences of condition dependence for mutation load [24] and adaptation to stressful environments [25,26], there has been little effort to test for this type of mutational plasticity in multicellular organisms. We investigated whether DNA repair ability is condition-dependent by exploiting the maternal repair system in D. melanogaster. When fertilized with mutagenized sperm, low-condition females were approximately 30% more likely than high-condition females to produce daughters carrying paternally derived X chromosomes that harbored recessive lethals. This result was consistent across two separate experiments.

The mutagen used in this experiment, MMS, causes lesions by alkylation of N atoms in the DNA [44]. These lesions can be repaired, without error, by excision repair. If a lesion is not repaired by excision prior to DNA replication, alternative error-prone repair mechanisms may be employed to remove the lesion, resulting in mutation [45]. Our data suggest that females in low condition are less able to efficiently repair DNA lesions without creating a mutation. This may be because low-condition females are more likely to employ error-prone repair pathways than are high-condition females or because low-condition females use error-free repair pathways less efficiently than do high-condition females.

We considered an alternative hypothesis based on sperm selection though there are several reasons to doubt this possibility. First, it is unlikely that the DNA lesions that lead to mutations causing lethality are the direct targets of selection, because only a very small fraction of genes are expressed in sperm [46]. However, the mutagen may cause physiological effects on sperm performance such that sperm exposed to heavier doses of mutagen would have both reduced performance and a higher likelihood of DNA damage at potentially lethal sites. In other words, lethal mutations may be eliminated via a correlated response to selection against other effects of the mutagen. Effective removal of X-linked lethal recessives through a correlated response would require either strong selection or a large covariance between the true target of selection (e.g., physiological effects of the mutagen) and the occurrence of X-linked lethal recessives.

Most importantly, sperm selection alone is not sufficient to explain the observed pattern. Rather, selection against mutagenized sperm must be stronger in high-condition females than in low-condition females. We tested this possibility and found no evidence for it. In fact, our data indicated that selection against mutagenized sperm was stronger in low-condition females. The reasons for this latter result are unclear but need not be adaptive. It is possible that the reproductive tract of a low-condition female is simply a harsher environment for sperm and imposes stronger selection. Finally, it is worth noting that, although we did detect significant selection against mutagenized sperm in the sperm competition experiment, this selection was weak. Moreover, this weak selection represents the selective difference between two extremes: mutagenized and non-mutagenized sperm. In contrast, any sperm selection that might have occurred in Experiments 1 and 2 would have been among sperm that experienced varying degrees of exposure to the mutagen, i.e., quantitative differences in exposure rather than qualitative differences as in the sperm competition experiment. Thus, any such selection in Experiments 1 and 2 would be expected to be even weaker than what we measured in the sperm competition experiment. In sum, we can infer that sperm selection was very weak in Experiments 1 and 2, and most likely worked in a direction opposite to the observed pattern with respect to sex-linked lethals.

Our data match the prediction expected under condition-dependent repair. Theory indicates that under most conditions, selection should favor reduced mutation rates in sexually reproducing organisms [47]. The direct costs of maintaining perfect DNA fidelity (i.e., the costs of perfect replication and error-free repair) are thought to prevent mutation rates from evolving to extremely low levels. This implies that repair mechanisms are expected to operate at a level that is somewhat costly. As has been discussed in other contexts (most notably for life history traits and secondary sexual characters), the expression of costly traits may often differ between individuals in high versus low condition [48–50]. Low-condition individuals may have higher mutation rates, not because selection favors more mutations, but simply because low-condition individuals cannot afford to invest as heavily in efficient repair.

We do not know whether the condition dependence in repair capacity reported here reflects condition dependence in mutation rates under natural conditions. If there is a relationship, we can speculate on how this would affect mutation load. Let us assume, as recent data suggest [17], that individuals in good condition have a mutation rate of *U_min_* = 1. In our study, females reared on the low-quality diet had 20%–30% lower fecundity and transmitted approximately 30% more recessive lethals than females reared on high-condition food. If we assume that a 30% reduction in condition translates into a 30% increase in mutation rate and that the relationship between condition and mutation rate is linear, we can calculate the mean fitness using previously developed theory [24]. Under these conditions, the mean fitness of a population at equilibrium is expected to be approximately 57% lower than expected if mutation was not condition-dependent (i.e., if *U* = 1 for all individuals regardless of condition). Obviously, this calculation is based on a number of untested assumptions, but nonetheless, it serves to illustrate that condition dependent mutation could have large effects on populations. Further work is required to explore these assumptions and evaluate the magnitude of condition dependence in mutation rate under natural conditions.

## Materials and Methods

### Sex-linked recessive lethal assay.

Experiment 1: females of high and low condition were produced through a larval diet manipulation. High-condition flies were created by rearing larvae on 7.5 ml of standard sugar-yeast-agar media at a low density (40 larvae per 8-dr [32-ml] vial). In the low-condition treatment, larvae were reared under identical conditions, but the media contained 25% of the standard concentration of sugar and yeast. Adult virgin females were collected within 8 h of eclosion. Both high- and low-condition adult females were held in vials containing standard media, but high-condition females were also given live yeast immediately. Females of both treatments received additional live yeast 3 d prior to mating. Females were mated to mutagenized males (described below) when they were 4 d (block 2) or 5 d (block 1) old. All of the females used were heterozygous for the balancer X chromosome *Basc* that is marked with the dominant eye mutation *B* (bar eyes). The *Basc* chromosome had been crossed into our standard large outbred population (Dah) more than ten generations prior to the experiment; the balancer chromosome was maintained in this stock by selection. The Dah outbred population was originally collected in 1970 in Dahomey (now Benin), West Africa. It has been maintained at large population size in various labs since that time and most recently in the current lab for over 3 y. Thus, the *Basc* heterozygous females used in this experiment had wild-type outbred genotypes other than the presence of the *Basc* chromosome.

Wild-type males (from the Dah population) were kept without access to food or water for several hours then exposed to sugar water with 1.5 mM MMS. The following day, males were transferred to recovery bottles for 2 h before allowing them to mate with the high- and low-condition females described above. Matings occurred in vials containing approximately 20 flies of each sex. The next day, females were transferred to individual vials to lay eggs for 1 d. All vials contained standard medium and live yeast. We counted the number of offspring emerging from these vials, allowing us to examine how the diet manipulation of mothers affected their production of offspring.

We used these F_1_ offspring to look for SLRL mutations. Our assay for SLRLs is very similar to traditional designs [38] and is shown in Figure 1. From each of the original *Basc* heterozygous females, four to eight (nonvirgin) *Basc/*X*_i_* daughters (F_1_), which had mated with their brothers prior to collection (see Figure 1 for details), were placed in individual vials to lay eggs so that their offspring could be scored. The symbol “X*_i_*” represents a paternally inherited (i.e., mutagen-exposed) X chromosome. Regardless of her mate, a *Basc/*X*_i_* female should produce two types of sons, *Basc/*Y and X*_i_*/Y, in equal frequency. However, if a recessive lethal mutation has occurred on this chromosome, then the *Basc/*X*_i_* daughter will be unable to produce wild-type sons (X*_i_*/Y).

If there is no recessive lethal on X*_i_*, then the expected frequency of wild-type males among the F_2_ progeny is 25%, assuming no viability differences among genotypes. If X*_i_* contains a recessive lethal (or near-lethal) mutation, then the frequency of wild-type males among the F_2_ progeny should be much less than 25%. We determined whether a given X*_i_* was likely to contain such a mutation by examining the observed frequency of wild-type males among a set of F_2_ progeny relative to the expectations if there was no mutation and if there was a mutation. Specifically, for each set of F_2_ offspring originating from a single F_1_ female, we calculated *R* = *L*_1/40_ */L*_1/4_. *L*_1/4_ is the likelihood (assuming a binomial distribution) of the observed offspring array if the true frequency of wild-type males among all possible sets of viable progeny of the family is 25%, i.e., the expected frequency if there is no mutation. Similarly, *L*_1/40_ is the likelihood of the observed offspring array if the true frequency of wild-type males among all possible sets of viable progeny of the family is 2.5% (as expected if the viability of wild-type males was approximately 10% of normal). A low value of *R* indicates it is unlikely that the X*_i_* in question contains a recessive lethal (or near lethal), whereas a high value of *R* indicates the opposite. When *R* ≥ 10, we classified the X*_i_* as carrying a recessive lethal; when *R* ≤ 0.1, we classified the X*_i_* as not carrying a recessive lethal. For intermediate values, 0.1 < *R* < 10, we were unable to clearly assign X*_i_* to either category, and these data were excluded.

Using this criteria, we calculated the frequency of lethal-bearing, paternally inherited X chromosomes transmitted by each of the original high- and low-condition females. Over 78,000 flies from 2,470 sets of F_2_ offspring were scored in Experiment 1. From these 2,470 sets of F_2_ offspring, we were able to classify 1,461 X*_i_* chromosomes as being *unlikely* to carry a recessive lethal (R ≤ 0.1) and 236 X*_i_* chromosomes as being *likely* to carry a recessive lethal (R ≥ 10); the X*_i_* chromosomes from the remaining 773 sets of F_2_ offspring were not classifiable (0.1 < R < 10). For these 1,461 + 236 = 1,697 classifiable X*_i_* chromosomes, the average number of F_2_ offspring upon which each classification had been based was 43.9 (standard error [SE] = 0.75). These 1,697 classifiable X*_i_* chromosomes had been transmitted by 552 parental generation females (283 high condition + 269 low condition), giving an average of 3.1 classifiable X*_i_* chromosomes per parental generation female. For each of these 552 parental generation females, we calculated the frequency of her classifiable X*_i_* chromosomes that were likely to carry a recessive lethal.

The average frequency of lethal transmission was compared between females from the two diet treatments. The true value of the difference in average frequency of lethal transmission was compared to a null distribution created by randomizing the data across treatments but within blocks; 10,000 randomizations were performed. The reported *p*-values for both Experiment 1 and 2 are for one-tailed tests because we had a clear a priori prediction about the direction of effect.

Experiment 2: several months later, we performed a second experiment that was very similar to the one described above except that mutagenized males were mated individually to experimental females, thereby suppressing the opportunity for sexual selection. In this experiment, females were 4 d old (block 1) or 3 d old (block 2) at the time of mating. Up to ten *Basc/*X*_i_* daughters were tested per female.

Over 130,000 flies from 4,857 sets of F_2_ offspring were scored in Experiment 2. From these 4,857 sets of F_2_ offspring, we were able to classify 2,648 X*_i_* chromosomes as being *unlikely* to carry a recessive lethal (R ≤ 0.1) and 291 X*_i_* chromosomes as being *likely* to carry a recessive lethal (R ≥ 10); the X*_i_* chromosomes from the remaining 1,918 sets of F_2_ offspring were not classifiable (0.1 < R < 10). For these 2,648 + 291 = 2,939 classifiable X*_i_* chromosomes, the average number of F_2_ offspring upon which each classification had been based was 41.6 (SE = 0.43). These 2,939 classifiable X*_i_* chromosomes had been transmitted by 595 parental generation females (309 high condition + 286 low condition), giving an average of 4.9 classifiable X*_i_* chromosomes per female. For each of these 595 parental generation females, we calculated the frequency of her classifiable X*_i_* chromosomes that were likely to carry a recessive lethal. As in Experiment 1, we used a randomization test to compare the average frequency of lethal transmission between high- and low-condition females.

To assess the total evidence for an effect of female condition on the rate of sex-linked lethal mutation, we used the weighted Z-transform method [39] to obtain a combined *p*-value from Experiments 1 and 2. Each study was weighted by its sample size. The unweighted Z-transform method provided a very similar result.

### Sperm competition experiment.

High- and low-condition females were created as described above. All of the females for this experiment were homozygous for a recessive *bw* ^−^ allele that causes brown eyes. Virgin females were mated to standard red-eyed males (homozygous wild-type at the *bw* locus) when they were 2 d (block 1), 3 d (block 2), or 4 d (block 3) old. After 2–4 h, males were removed, and females were placed in individual vials to lay eggs for 4 d. Females that did not produce viable eggs during this period were discarded. Females were then mated to *bw^−^/bw^−^* males. Prior to mating, these males had either been mutagenized with 1.5 mM MMS as described above or put through an equivalent sham treatment without any mutagen. After 1 d, the males were discarded, and the females were transferred to new, individual vials to lay eggs for 2 d (egg-laying vial 1). Females were then flipped into new vials for another 2 d of egg laying (egg-laying vial 2). Offspring emerging from both egg-laying vials 1 and 2 were scored for eye color to determine paternity.

Because of the strong last-male precedence in *Drosophila*, it is likely that females producing no brown-eyed offspring had not mated with the second male; these females were excluded from the analysis. We also excluded vials containing fewer than ten offspring. Data were analyzed with a generalized linear mixed model using PROC GLIMMIX in SAS with a logit link function and a binomial error structure where female condition and male mutagen treatment were included as fixed factors, and block and vial were included as random effects.

In addition to the analysis above, we also used a likelihood framework to model the proportion of offspring sired by the second male, *P*_2_, as a function of block, female condition, and selection against mutagenized sperm. Separate parameters modeled the strength of selection occurring in low-condition females and in high-condition females, though the model can be constrained so that selection is the same in both types of females. This analysis ignores variation among vials and so may underestimate the uncertainty in parameter estimates. Nonetheless, the parameters are easily interpreted biologically and thus have heuristic value.

Specifically, the likelihood analysis worked as follows. Let *X* be an indicator variable specifying whether a female's second mate had been mutagenized (*X* = 1) or not (*X* = −1). For low-condition females, the proportion of offspring sired by the second male is modeled as *P*_2,low_(*X*) = *k_i_*(1 − *f*)(1 − *X t_low_*); for high-condition females, *P*_2,high_(*X*) = *k_i_*(1 + *f*)(1 − *X t_high_*). The parameters *k_i_*, *f*, *t_low_*, and *t_high_* describe the effect of different factors on *P*_2_: *k_i_* is approximately the average level of *P*_2_ in block *i* (*i* ∈{1, 2, 3}), *f* is the effect of female condition on *P*_2_, *t_low_* is the disadvantage of mutagenized sperm in low females, and *t_high_* is the disadvantage of mutagenized sperm in high females.

Let *m_ij_* be the observed number of offspring from female *j* in block *i* that were sired by the second male; let *n_ij_* be the total observed number of offspring from this female. If the true expected siring success of the second male is *p*, the probability that *m* out of the *n* offspring produced by a female will be sired by the second male is given by the binomial distribution, Pr(*m*|*n,p*) = (*n*!/(*m*!(*n-m*)!))*p^m^*(1 − *p*)*^n-m^* where *p* is *P*_2,low_(*X*) or *P*_2,high_(*X*), as appropriate depending on the female's condition and the mutagen status of her second mate.

Considering the data from all females, the negative log likelihood of parameter set **x** = {*k*_1_, *k*_2_, *f, t_low_, t_high_*} is given by

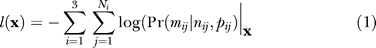
where *N_i_* is the total number of females in block *i*. A modified simulated annealing procedure, originating from 25 different random parameter combinations, was used to find the parameters that minimized the value of *l*(**x**), i.e., the maximum likelihood parameter estimates.

We calculated the maximum likelihood of the unconstrained model and the maximum likelihood of a constrained model in which the disadvantage of mutagenized sperm was assumed to be the same in both high- and low-condition females, i.e., *t_low_* = *t_high_*. The maximum likelihood parameters of the unconstrained model gave *l*(**x**_max, unconstrained_) =1,587.8, whereas for the constrained model, *l*(**x**_max, constrained_) =1,596.4. The unconstrained model had a significantly higher likelihood than the constrained model (likelihood ratio test, *χ* ^2^ = 17.4, *df* = 1, *p* = 3 × 10^−5^) indicating that strength of sperm selection differed significantly between high- and low-condition females. The maximum likelihood parameter estimates for the constrained model were *k*_1_ = 0.96, *k*_2_ = 0.93, *k*_3_ = 0.95, *f* = −0.002, *t_low_* = 0.019, and *t_high_* = 0.003; the latter two values indicating selection against mutagenized sperm is stronger in low-condition females than in high-condition females. The standard population genetic parameterization of selection *s* comes from the reduction in fitness of the less fit type relative to the more fit type, i.e., *w_less_* = (1 − *s*)*w_more_*. In this case, the less fit type are mutagenized males and the more fit type are non-mutagenized males. The parameter *t* is related to *s* by the equation *s* = 2*t*/(1 + *t*). This relationship was used to produce the values of *s_L_* and *s_H_* given in the Results.

## Abbreviations

MMS: methyl methanesulfonate
SLRL: sex-linked recessive lethal
